# Cutaneous Leishmaniasis in Tigray, North Ethiopia: Community Awareness, Perceptions, Treatment-Seeking, and Prevention Practices in Disease Endemic Areas

**DOI:** 10.3390/tropicalmed11070176

**Published:** 2026-06-27

**Authors:** Shewaye Belay Tessema, Afework Mulugeta Bezabih, Helen P. Price

**Affiliations:** 1 Department of Parasitology, Faculty of Medical Laboratory Sciences, College of Health Sciences, Mekelle University, Mekelle P.O. Box 1871, Tigray, Ethiopia; shewayebelay@yahoo.com; 2 School of Public Health, College of Health Sciences, Mekelle University, Mekelle P.O. Box 1871, Tigray, Ethiopia; afework.mulugeta@gmail.com; 3 School of Life Sciences, Keele University, Newcastle-under-Lyme ST5 5BG, Staffordshire, UK

**Keywords:** cutaneous leishmaniasis, knowledge, attitudes and practice, Tigray, Ethiopia, disease awareness

## Abstract

Cutaneous leishmaniasis (CL) is highly prevalent in northern Ethiopia but data on community knowledge, attitudes, and health-seeking behaviours remain limited. A cross-sectional survey was conducted between November and December 2022 in CL-endemic areas of Tigray using mixed sampling and a structured questionnaire administered to 512 households. Knowledge of CL transmission was poor: only 1% correctly identified sand flies as the vector, while 25% believed the disease was genetically acquired. Approximately 67% of participants perceived CL as stigmatizing, and 63.3% reported a preference for traditional or local treatments over biomedical care. Knowledge levels were higher among rural residents and in households with prior CL experience. Gender and education were significantly associated with treatment-seeking and prevention practices, and participants from households with previous CL episodes reported better practices overall. Despite this, most participants demonstrated limited knowledge, unfavourable attitudes and suboptimal treatment-seeking and prevention behaviours. These findings highlight a disconnect between high disease burden, perceived seriousness and stigma, and limited understanding of transmission and prevention. Targeted, community-based health education interventions are needed to improve awareness of transmission, reduce stigma, and enhance access to effective treatment in CL-endemic settings.

## 1. Introduction

Cutaneous leishmaniasis (CL), one of the vector-borne neglected tropical diseases (NTDs), is caused by infection with protozoan parasites of the genus *Leishmania* and transmitted via the bites of infected female sand flies [1]. The disease is endemic in more than 90 countries worldwide. There are approximately 0.7 to 1.2 million new cases annually across the globe and around 350–430 million people are estimated to be at risk of infection [2,3,4,5]. Furthermore, about 40 million people globally are living with CL scars from past infections [6] and both active disease and scars from previous infections can lead to significant stigma of individuals and their communities [7]. However, there is substantial underreporting of this disease, with only 200,000 cases per annum being reported to the World Health Organization (WHO) [3,8,9].

In Ethiopia, reports of CL date back at least to the early 1900s, with the first case reported from the northern part of the country and described as “Oriental sore in Agamé (Abyssinia)” [10]. Since then, the disease has been reported from the highlands of the country and over 170 districts are suspected to be endemic [11]. In Ethiopia, CL presents in three clinical forms, localized (LCL), diffused (DCL), and mucocutaneous (MCL) [12], of which MCL and DCL present significant therapeutic challenges [13]. Studies have estimated that 20,000 to 50,000 people per year are infected in Ethiopia [2,14]. However, in 2022, only 913 CL cases were reported to the WHO [8], suggesting a gross underreporting of the disease in Ethiopia. The primary etiological agent of CL in Ethiopia is *L. aethiopica* [12]. Transmission is driven by the sand fly vectors *Phlebotomus longipes* in the north of the country and *P. pedifer* in the south, which are both zoophilic and anthropophilic. A major reservoir host for *L. aethiopica* is the rock hyrax (*Procavia capensis*), a rodent which resides in rocky mountainous regions in close proximity to the sand fly vector [12].

We recently published findings from a cross-sectional household study in Tigray, where we identified a high prevalence of CL particularly in children and the presence of the more severe forms, MCL and DCL [15]. We found that 12.7% of participants had clinical signs of CL, with active lesions (4.5%) or characteristic scars (8.2%). Cases were clustered in highland zones (93% at 2000–3000 m altitude above sea level) and risk factors included age, outdoor sleeping, poor housing, and proximity to caves [15]. Our research indicates that CL is a major health issue in the region and that efforts need to be increased to address the disease.

A critical factor in addressing the burden of infectious diseases is to improve community awareness, behaviours, and prevention practices [16,17]. Knowledge, attitude, and practice (KAP) evaluation surveys provide fundamental evidence for health promotion campaigns, enabling health education messages to be specifically tailored to address gaps in public knowledge and awareness. Several studies indicate a direct relationship between community awareness on CL and effectiveness of control strategies [18,19]. Understanding and practices around infectious diseases such as CL are heavily influenced by socio-cultural settings and widely vary among communities across different regions of the country; however, only a small number of KAP studies have been conducted in CL-endemic regions in northern Ethiopia [20,21,22] and community health education towards prevention and control of the disease is lacking [12]. Furthermore, the first line biomedical treatment for CL in Ethiopia is multiple injections with pentavalent antimonial drugs, which can present major challenges regarding administration and treatment access for remote rural communities.

In the present study, we evaluated the knowledge, attitude, and practice (KAP) and treatment-seeking behaviours and prevention practices of communities located in CL-endemic districts of the Tigray region in order to inform disease control interventions. This work was carried out as part of a wider interdisciplinary study, ECLIPSE, which aimed to improve the patient journey and reduce stigma for people living with CL in endemic regions of Brazil, Ethiopia, and Sri Lanka.

## 2. Methods

### 2.1. Study Setting

The Tigray region is located in the northern part of Ethiopia between 12O 15′ N and 14O 57′ N latitude and 36O 27′ E and 39O 59′ E longitude. The Tigray region has 7 administrative zones namely Central, Eastern, Mekelle, North Western, Southern, South Eastern, Western, and Mekelle; 52 districts (locally known as woredas); and 799 sub-districts or kebeles (locally, Tabias). A Tabia is the smallest administrative unit in the region which consists of 8 to 10 small villages (called kushets) and a kushet comprises on average from 150 to 250 households. The topography of Tigray consists of high plateaux and mountains with much of the land lying between 1000 and 3900 m above sea level altitude. This study was conducted in seven CL-endemic districts found in three zones of Tigray, namely Ganta Afeshum, Gulomekeda, Hawzen and Saesie Tsaeda-emba located in the Eastern zone, Degua Temben and Enderta in the South Eastern zone, and Emba-alaje situated in the Southern zone.

### 2.2. Study Design and Study Period

A cross-sectional survey was carried out between November and December 2022, in seven districts located in three zones of Tigray, northern Ethiopia.

### 2.3. Sample Size

The minimum sample size required for this survey was calculated according to the WHO’s practical manual for sample size determination in health studies [23]. As previous data towards the level of community awareness and practices related to CL in the study areas were unavailable, we assumed a 50% level of awareness and practices in the targeted area study communities. The sample size was calculated using a single population proportion formula, assuming 95% CI with 0.05 margin of error and a 1.3 design effect.

*n* = [Z^2^ × P(1 − P) × D]/e^2^ = [(1.96)^2^× 0.5(1 − 0.5)1.2]/(0.05)^2^ = 499

The minimum sample size required for this study was 499 respondents using the following assumption: *n* = the number of study subjects (household heads), Z is a critical value (1.96) at 95% confidence level, P = anticipated population proportion (50%), D = design effect and e = margin of error (5%). To the calculated minimum sample size (*n* = 499), 5% of non-response rate was added and the total sample size was determined to be 524 individual participants.

### 2.4. Sampling Procedure

**Figure 1:**
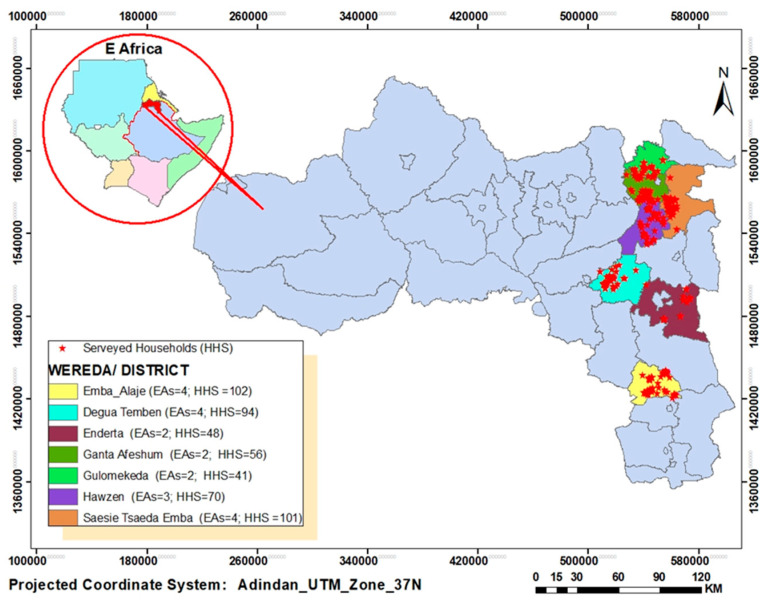
Study districts, number of Enumeration Areas (EAs) and number of surveyed households (HHs). The red circles represent participating households in the survey. The base map used is obtained from an openly available source at https://open.africa/dataset/ethiopia-shapefiles (accessed on 1 March 2026).

Between November and December 2022, a cross-sectional survey was conducted among communities living in seven districts located in three zones of Tigray. In the first stage of sampling, CL-endemic districts [15], namely Ganta Afeshum, Gulomekeda, Hawzen and Saesie Tsaeda-emba districts (from the Eastern zone); Degua Temben and Enderta (South Eastern zone); and Emba-alaje district from the Southern zone. In the next stage, utilizing data from the Central Statistics Agency (ECSA, 2012) [24], cluster Tabias or Enumeration Areas (EAs) were selected using probability proportionate to size (PPS). Accordingly, Enumeration Areas (EAs) from Degua Temben (*n* = 4), Emba-alaje (*n* = 4), Enderta (*n* = 2), Ganta Afeshum (*n* = 2), Gulomekeda (*n* = 2), Hawzen (*n* = 3), and Saesie Tsaeda-emba (*n* = 4) were included. Larger population size Tabias or Enumeration Areas (EAs, *n* = 21) were included so as to reach the number of households (HHs; *n* = 25) required to achieve the overall sample size [(21 clusters 25 households) = 524 HHs] needed in this study (Figure 1). Then, a simple random sampling was used to select households and finally, the most knowledgeable family member or the head of a household (one individual per household) was approached for an interview. Both male and female adults aged 18 years and above were eligible in this study.

### 2.5. Data Collection Tools and Procedures

Data were collected using a structured questionnaire adopted from similar studies [16,18,21,25], pre-tested on fifteen individuals recruited from households in the seven study districts. The questionnaire was designed to obtain information on participants’ socio-demographic characteristics; knowledge, attitude and practices (KAP); and treatment-seeking behaviour towards CL. The questionnaire was developed in English and then translated into the participants’ native language (Tigrigna), and the local name for CL (Guzwa, ጉዝዋ) was used to refer to the disease. The questionnaire was composed of four sections.

Section 1 of the questionnaire consisted of information on participants’ socio-demographic characteristics. Section 2 contained questions on knowledge about CL that were designed to identify the primary sources of information used, to understand participants’ ability to identify the disease and explore their knowledge of aspects of CL, including signs and symptoms, local name of the disease, transmission mechanisms, treatment options, modern healthcare facilities, the existence of hyrax (the main reservoir host) and its relation with CL, and whether CL can be prevented. Section 3 contained questions designed to determine the participants’ attitudes towards CL as a health concern, stigma, whether the disease is curable by treatment, feelings on seeing people with CL, primary care provider choices, and attitudes towards use of biomedical treatment for CL. Section 4 consisted of questions designed to evaluate the treatment-seeking and prevention practices of the participants, such as primary healthcare options for active CL, reasons to choose a care provider, and distance from healthcare facilities. Additional questions were included to assess potential risk factors, such as occupation, time spent working outside (including overnight stays), use of hyrax dung as fertilizer, outdoor sleeping habits, and preventive measures.

Questionnaires were administrated by a team of trained and experienced senior health professional data collectors who had previously been involved in leishmaniasis-related household surveys. Data was primarily collected from the head of a household; when the head was not available, any responsible adult above 18 years selected by the family as the most knowledgeable person of the household was approached by the survey team for participation in the survey.

### 2.6. Scoring Methods

Scores for the KAP of respondents were developed using methods described in previous studies [16,20]. In brief, from the KAP questionnaire, a composite score of each question or item was calculated for each participant where each correct (or positive) response was assigned a score of 1 and each incorrect or unsure response was assigned a score of 0. The total scores were further dichotomized based on the overall scores of each item. The total knowledge scores ranged from 0 to 9, and scores between 0 and 4 were categorized as poor knowledge, while scores between 5 and 9 were considered indicative of good knowledge, as described previously [9]. Similarly, the total attitude scores ranged from 0 to 6; scores between 0 and 3 were categorized as a negative attitude, while scores between 4 and 6 were considered to denote a positive attitude. With regard to treatment-seeking behaviour and prevention practices, scores ranged from 0 to 12. Scores between 0 and 6 were considered to indicate poor prevention practices, while scores between 7 and 12 were considered to be good prevention practices. While this scoring method has been used previously, we acknowledge that there are some limitations to this approach and that some areas of knowledge have greater relevance to the affected communities than others.

### 2.7. Data Analysis

The collected data were entered into EPI Info version 20.1.14 statistical package (Centers for Disease Control and Prevention) and exported to SPSS version 25.0 (SPSS, IBM Inc., Chicago, IL, USA) for statistical analysis. Descriptive statistics such as frequency, percentage, and mean (standard deviation) were used, where applicable, to describe the KAP components with the explanatory (independent) variables. A chi-square test was used to examine the associations between KAP scores and the explanatory variables such as study area settings, age, gender, education level, occupation, and CL infection episode in a household. A multivariate logistic regression analysis was performed to identify the significant predictors of good knowledge on CL, positive attitude, and good practices towards CL treatment-seeking behaviour and prevention. Variables with a *p* value of ≤0.25 in the chi-square test were included in the logistic regression analyses. Adjusted odd ratios (AORs) and their corresponding 95% confidence intervals (CIs) were calculated based on the final models. The significance level for all tests was set at *p* < 0.05.

## 3. Results

### 3.1. Socio-Demographic Characteristics of the Study Participants

A total of 512 individuals from different households (52.3% male and 47.7% female) participated in the study, with a 97.7% response rate. Of these, 70.5% were between 18 and 40 years old. More than half (52.3%) of the participants were from the eastern zone, a majority were rural inhabitants, and over three quarters (79.3%) were farmers by occupation. With respect to education level, 69.9% of the participants had attended modern schools and 30.1% of them had not received any formal education. Of the 358 educated participants, 39.4% and 29.1% had completed primary (1–6th grades) and secondary (7–10th grades) school levels, respectively. Only seven (1.4%) respondents had completed college and university education level. Out of all participants, about 61% acknowledged that one or more CL episodes (at least one case identified through symptoms or diagnosed at healthcare) had occurred within their household during the period of the last five years (Table 1), showing that this disease is highly endemic in the region, in agreement with our recent epidemiological study [15].

### 3.2. Knowledge About Cutaneous Leishmaniasis

In the present study, a large majority of participants, 97.3% (498/512), had some prior knowledge about the disease. The main sources of information around CL were household family members who had experience of the disease, and neighbours. Most participants (96.8%) described the disease using its local name “Guzwa” or ጉዝዋ (the local name for CL, in Tigrigna language), while only 16 (3.2%) respondents had heard of the term “leishmaniasis”, the scientific name for the disease. A majority of the participants (71.5%) responded that skin lesions were the main sign of CL and over 95% described that the lesions appear on the face (Table 2).

While the clinical signs of the disease were recognized, very few participants knew the biological cause and transmission route for CL. Only six (1%) participants were able to name the sand fly vector and 21.1% did not name any transmission mechanisms. While 32.3% and 17.5% of the participants named bats and moths/butterflies as being disease vectors, respectively, 24.9% believed that CL was a genetically acquired disease. A majority of participants (73.6%) had seen rock hyrax (the major reservoir host for *Leishmania* in this region) in their localities; however, only twelve respondents (3.2%) knew that the hyrax was linked to risk of CL.

In response to questions on treatment priorities, traditional healers (herbalists) were the primary choice for the majority (62.9%) of participants followed by religious healers/faith remedies (14.1%). Over 67% of the participants did not know about biomedical treatments for CL and about two thirds (68.2%) did not name any CL prevention measures. Based on the overall scoring analysis, 219 (42.8%) participants had what we defined as a relatively “good” level of knowledge about CL (with scores of 5–9), while 293 (57.2%) had a “poor” level of knowledge (scores of 0–4) towards the disease (Table 2).

### 3.3. Attitudes Towards CL, Its Treatment, and Care Provider Priorities

Most participants (96%) perceived CL to be a serious public health concern in their locality. Over 67% of the participants responded that CL is a stigmatizing disease. However, when participants were asked a subsequent question about their personal feelings when meeting people with a CL lesion, most of the participants (87.1%) responded that they felt uncomfortable.

Traditional healers (herbalists) were the primary choices for 73.6% of the participants and faith healers (religious remedies) were named as the first choice by 16.4%. Formal healthcare settings (defined here as community health posts, health centres, and hospitals at all levels) were the first point of contact for only 45 (10%) respondents. Based on the scoring analysis, 187 (36.5%) participants had a positive attitude toward CL (scores of 4–6 for selected questions), while 325 (63.5%) had a negative attitude (scores of 1–3) (Table 3). The majority of respondents (87.8%) perceived CL to be a curable disease but only 27.3% stated that CL could be prevented, with 68% replying that they did not know.

### 3.4. Treatment-Seeking Behaviours and Practices

Most participants (83%) stated that people used homemade herbal remedies as the primary method of care, while 63.3% used traditional remedies from a healer (herbalist), 13.7% used religious healing (faith remedies), and 17.4% of the participants used cauterization of lesions using very hot metal, a practice which is highly likely to cause pain and skin damage.

About 27.3% (136/498) of the participants stated that they had used CL treatments at formal healthcare facilities, either for themselves or members of their household/families. Of those (*n* = 136) who had used a formal CL healthcare facility, about 85% had used one or more traditional remedies before receiving biomedical treatment, 66.9% had delays of 1–6 months, and 14.7% had delays longer than 6 months before receiving this form of treatment. Regarding the nearest formal CL healthcare facility, 60.4% of participants stated this was 61–90 km away from home and 26.8% responded that it was 30–60 km away.

Participants were asked about situations where people prefer to seek formal healthcare for CL treatment; interestingly, this question revealed a concept of gendered lesions: 237 participants (47.6%) stated that they would seek healthcare when the CL lesion is a “male” type, wording used to describe a more severe type of lesion that does not heal easily (potentially MCL or DCL types of the disease). Some respondents also mentioned a “runner” or wet type of lesion. In addition, 78 (15.7%) indicated that they would seek healthcare when the wound failed to heal using traditional treatments (Table 4). Based on the scoring analysis outcome, while 173 participants (33.8%) had a good level of treatment-seeking behaviour and practices towards CL, 339 (66.2%) had a poor level of practices (Table 5).

Almost half (45.3%) of the participants reported that they were commonly engaged working in fields from early morning until late evening, and 23.1% of the participants worked in fields during both daytime and at night, potentially increasing the risk of sand fly bites. Over half (58.2%) of the participants had outdoor sleeping habits and over one third (37.9%) of them used hyrax dung as fertilizer, potentially attracting sand flies. These were identified as key risk factors in our epidemiology study in Tigray [15].

Over 71% of the participants did not apply any known CL preventive measures; however, about 28.6% (140/489) of the participants stated one or more mechanisms. Of those, 28%, 10.6%, and 7.4% of the participants mentioned using bed nets, improving environmental hygiene, and wearing long sleeves/trousers, respectively, as preventive measures for the disease. Based on the scoring analysis outcome, while 173 (33.8%) participants had a good level of prevention practices towards CL, 339 (66.2%) had a poor level of prevention practices (Table 5).

### 3.5. Factors Associated with Knowledge About CL

Males were less likely to have good knowledge of CL compared to females (*p* = 0.004) (Table 6). Farmers were also less likely to have good knowledge than those in other occupations (*p* = 0.021), while rural residents were 1.6 times more likely to have good knowledge than urban or semi-urban participants (*p* = 0.049). Participants from households with a previous CL case were over ten times more likely to have good knowledge (*p* < 0.001), representing the strongest association observed.

After adjustment for confounding, gender, occupation, rural residence, and prior household CL experience remained independently associated with knowledge, with prior CL exposure showing the strongest effect (AOR 10.19). In contrast, age and education were not significant in the adjusted model, suggesting that their crude associations were explained by other correlated factors (Table 6).

### 3.6. Factors Associated with Participants’ Attitude Towards CL

Occupation and household setting were significant factors associated with attitudes towards CL Table 7; *p* < 0.05). Participants engaged in farming were 1.6 times more likely to have an unfavourable attitude compared with those in other occupations, while those from urban and semi-urban areas were around 2.2 times more likely to have a favourable attitude than rural residents (Table 7). These findings contrast with the higher levels of knowledge observed among rural populations (Table 6).

Together, these results suggest that knowledge and attitudes could be influenced by different underlying factors or combinations of competing factors, and that greater disease awareness alone does not necessarily translate into more favourable perceptions of affected individuals.

### 3.7. Factors Associated with Treatment-Seeking and Prevention Practices

Male participants were about 1.5 times more likely to have poor treatment-seeking and prevention practices compared to female counterparts (*p* = 0.044). Participants with some formal education were 2.6 times less likely to have poor practices than those with no formal education (*p* < 0.001). The likelihood of good practices was also approximately threefold higher among participants from households with previous or current CL cases (*p* < 0.001), representing the strongest association observed (Table 8).

These findings are consistent with the higher levels of knowledge observed among educated participants and those with prior household CL exposure (Table 6), suggesting that both formal education and lived experience play key roles in shaping health behaviours. However, the persistence of poor practices among a substantial proportion of participants indicates that knowledge alone is insufficient, and that structural barriers such as access to healthcare and reliance on traditional treatments may also influence behaviour.

## 4. Discussion

### 4.1. High Burden of CL in Tigray

The current study revealed that a large proportion (61%) of participants in our study sites had experienced one or more CL episodes within their household during the last five years. This agrees with our recent epidemiological study in Tigray [15] in showing that this disease is highly endemic and a serious public health challenge in the region. This remains in stark contrast to the low numbers of cases officially reported annually to the WHO from Ethiopia [8]. The results from the current study also revealed that, despite the relatively high prevalence of the disease, many participants had a poor level of knowledge relating to CL transmission and negative attitudes towards people with CL lesion. Over 67% of the participants believed that CL is a stigmatizing disease and over 87% acknowledged that they felt uncomfortable when meeting people with active lesions, indicating that stigma associated with this disease is common in this region. The study findings also showed that many participants had a poor level of treatment-seeking and prevention practices. These findings are likely to be deeply rooted in the neglected nature of CL in government health policies and a lack of available information on disease control measures and healthcare options.

### 4.2. Knowledge of CL in Communities in Tigray

Using the KAP scoring system described previously [9], we designated scores to a subset of questions and separated the responders into those with “good” and “poor” levels of knowledge. While having some limitations, this method allowed for the analysis of factors contributing to the overall levels of knowledge around CL. Around 42.8% of the current participants were defined as having a “good” level of knowledge about CL. This finding was higher than in comparable studies in the Delanta district, northeast Ethiopia (where 27.65% of respondents had good knowledge) [26]; the Wolaita zone, southern Ethiopia (19%) [20]; the Kutaber district, northeast Ethiopia (22.9%) [22]; and in the Shara’b district, southwestern Yemen (22.3%) [16]. However, the current finding was lower than the knowledge level (67.6%) found in Ochello, Gamo Gofa zone, southern Ethiopia [18] and a study among communities in hyperendemic areas in western Yemen, where 51.2% of participants had overall “good” knowledge about CL [9]. This knowledge-level discrepancy could be due to multiple factors, including differences in study areas and methodologies, variations in magnitudes of CL prevalence among investigated countries, and the socio-demographic and socio-cultural variations in each studied population.

In the current study, the highest scores were achieved for questions around the clinical signs of the disease, which is a reflection of the high prevalence and visibility of lesions. A large majority of participants were familiar with the disease, had seen cases in their community, and described the condition using a common local term “Guzwa” (a name for CL in Tigrigna language), while only very few had heard the scientific name of the disease, “leishmaniasis”. Our findings are consistent with studies carried out in other CL-endemic regions including Amhara, Ethiopia [22], the Volta region of Ghana [16], and southwestern Yemen [27], and in contrast to a study in a non-endemic area of Alexandria, Egypt, where most participants (90%) had never encountered CL [28]. Further, we found that households who had experienced at least one case of CL during the last five years were significantly more likely to have a good knowledge level.

While most of the participants in our study showed the ability to recognize the clinical signs of CL, they had a very poor level of knowledge around its transmission mechanisms and only six participants (1%) mentioned the sand fly vector by name. This finding is comparable with a similar study conducted in the Wolaita zone, southern Ethiopia, where none of the participants had heard of the sand fly [20] and is also consistent with other similar studies conducted in Ethiopia [18,21]. Sand flies are very small insects (typically 1 mm in length) and difficult to see without magnification, which is likely to be a key contributing factor to this knowledge gap, in addition to a lack of locally available public health information on the disease. While knowing the specific vector species might not be essential for community members, wide recognition that CL is spread by an insect vector will be critical for the successful implementation of disease prevention strategies.

Participants in the current study had high levels of misconceptions around transmission mechanisms of the disease: one quarter of the current participants considered CL to be a genetically acquired disease, about one third considered bats to be the disease vector, and about one fifth of the participants believed that butterflies or moths could be responsible for spreading the disease. These findings are similar to previous studies conducted in Ethiopia. For example, 61.6% of study participants in the Kutaber district had misconceptions on the mode of transmission [22] and 19.5% of participants in a study in the Delanta district responded that CL is transmitted via bat urine [26]. However, unlike the findings in our study, a similar KAP study in central Iran found that 97.9% of the participants had a theoretical knowledge of the sand fly vector [29]. The differences could be due to variations in socio-demographic factors, including the study settings and education level of participants and also the availability of accurate information on the disease. The study in Iran was institution-based and conducted with student participants [29], whereas the current study was conducted in CL-endemic community settings where 30% of participants had not received formal education.

A recent study in Sri Lanka [30] took a different approach to knowledge around CL, defining “CL disease awareness” through a public health lens and incorporating three fundamental elements that are important for affected communities to know. These were: (1) being aware of the disease name or local name; (2) being aware that the disease is spread by a sand fly vector; and (3) being aware that the disease is characterized by a long-lasting skin lesion. Using these criteria, the study in the Anuradhapura district (a CL-endemic region) identified that only 3.5% of participants had CL disease awareness, with very low scores for questions on two of these elements, transmission mode and recognizing clinical signs [30]. While many CL cases in Sri Lanka present with a single lesion localized on the limbs [31], our recent epidemiological study showed that the majority of cases in Tigray are found on the face and include more severe forms such as DCL and MCL [15], which may help to explain the differences in community awareness of clinical signs between the two geographical regions.

### 4.3. Attitudes Around CL in Tigray

In the current study, only 36.5% of participants had an overall favourable or positive attitude towards individuals with CL. This finding is comparable with earlier studies undertaken in northeast Ethiopia and studies in western Yemen [9,26]. Another study carried out in the Kutaber district of Ethiopia showed even greater effects, with only 18.2% of the study participants having a favourable attitude towards people showing signs of the disease [22].

Over two-thirds of the participants in the current study perceived CL to be a stigmatizing disease. A number of other studies have described the different types of stigmas associated with CL (reviewed by the authors [7]). Stigma is highly context-specific and has been linked to a fear of contagion and social rejection. Affected individuals may anticipate avoidance, experience shame, and internalize fear of disfigurement and others’ reactions, while also experiencing direct discrimination and exclusion [7,32,33,34]. In studies carried out in Morocco and Tunisia, CL has been linked to intense psychosocial distress, while low disease awareness and fear of person-to-person transmission can lead to discrimination and rejection [35,36,37]. These effects are strongly gendered, with women disproportionately affected due to facial scarring, undermining perceived beauty and marriage prospects, leading to greater social exclusion and constraints on participation and care-seeking [38,39]. The stigma associated with CL may also exacerbate health outcomes and influence the educational attainment of children [7].

A conceptual framework developed from a study in rural Sri Lanka highlights the complex drivers of CL-associated stigma [32]. Consistent with this and previous evidence, the high levels of perceived stigma observed in the current study are likely to be associated with poor understanding of disease transmission, local beliefs and misconceptions, and fear of the disease and its visible effects. However, our findings also suggest that unfavourable attitudes are not solely explained by low levels of knowledge, as rural participants, who demonstrated relatively higher knowledge, were more likely to report negative attitudes towards CL (Table 6 and Table 7). A substantial proportion of participants believed that CL is genetically acquired, which may influence social interactions, including relationships and marriage. In addition, stigma may be underestimated when assessed through direct questioning alone. When asked about personal reactions, the majority of participants reported feeling uncomfortable when encountering individuals with CL lesions, suggesting that enacted and internalized stigma may be more pervasive than indicated by reported attitudes.

The stigma associated with CL may lead to social discrimination, isolation, and reduced care-seeking, and can exacerbate health outcomes and affect educational attainment, particularly among children [7,32,33,34]. This study forms part of the wider ECLIPSE program, and further qualitative work will explore the complex social and cultural drivers of CL-related stigma in greater depth.

### 4.4. CL Treatment-Seeking Behaviours

Our multivariate analysis identified gender, education, and prior household CL experience as significant predictors of treatment-seeking and prevention practices (Table 8). Male participants were more likely to report poor practices, while formal education was strongly associated with improved behaviours, suggesting that both gendered roles and access to information influence health decision-making. In addition, participants from households with previous or current CL cases were significantly more likely to report good practices, indicating that lived experience of the disease plays a critical role in shaping behaviour.

These findings align with the associations observed for knowledge (Table 6), where prior CL exposure and education were also key determinants, highlighting a consistent pathway linking experience and awareness to behavioural outcomes. However, despite these associations, a substantial proportion of participants continued to demonstrate poor treatment-seeking and prevention practices, suggesting that knowledge alone is insufficient. Structural barriers, including distance to healthcare facilities, cost, and reliance on traditional healers, are likely to mediate this relationship and limit translation of knowledge into practice.

Our study showed that only 34% of participants had a good level of treatment-seeking behaviour and prevention practices, which is comparable to a similar study carried out in southern Ethiopia [18]. However, this is higher than a KAP study undertaken in western Yemen, where only 16.3% of the study participants had good levels of treatment-seeking and prevention practices [9]. This is heavily influenced by the prolonged civil war in Yemen which brought the public health system to collapse [9,40], therefore severely impacting healthcare access for CL and other chronic health conditions.

Likewise, over two-thirds of the present study participants had a poor level of formal healthcare-seeking behaviour for CL. The recent war in the Tigray region of northern Ethiopia (2020–2022) brought unimaginable humanitarian crisis and enormous damage to the health system in the region [41]. About 80% of the primary hospitals and 86% of the secondary and tertiary hospitals including the CL care provider hospitals were fully or partially damaged and/or vandalized/looted during the war and the majority of the health facilities became non-functional [42]. This is likely a key factor in the healthcare-seeking behaviours seen in affected communities.

Our findings showed that over 63% of participants preferred to seek remedies from traditional healers (herbalists) rather than formal CL healthcare facilities. This could be due to several factors, including local beliefs, low awareness about biomedical treatments available, and poor access to facilities. These findings are consistent with many community-based studies in Ethiopia: traditional remedies were the primary choice for 67.6% of participants in the Gamo-Gofa zone [18], 68.3% of participants in the Amhara region [21], and about 77% of participants in the Wolaita zone [20]. A study in Nigeria also found that participants preferred to seek CL treatments with traditional healers rather than formal healthcare services [43]. It is important to recognize that traditional healers have an important role within many African communities and often act as the first point of contact for health issues. However, we found evidence in our study of high-risk practices such as burning lesions with hot metal, which could cause substantial skin damage and scarring.

The current findings revealed that religious/faith healers (locally named “Tsebel”) offered traditional treatments specifically called “Tsebel Senbet Senabti”, which were among the most common traditional methods used to treat CL in the study sites. These findings are consistent with previous studies in southern Ethiopia [20]. Moreover, over 60% of the current participants acknowledged that the nearest formal CL healthcare facility is located approximately 61 to 90 km away from home. Most formal CL healthcare in Ethiopia, including in Tigray, is centralized and located in regional or zonal capital cities [44], a long distance from endemic areas. Decentralization of the current CL treatment facilities to the disease endemic localities could be one of the most important measures needed to improve biomedical treatment access for the most affected communities.

### 4.5. Practices Around CL Prevention

Our findings showed that most participants had no knowledge about CL prevention mechanisms, which is consistent with poor levels of understanding around the mode of disease transmission. A very small proportion of participants mentioned bed nets or other preventive methods. These findings are consistent with similar studies conducted in southern Ethiopia and western Yemen [9,20]. About three quarters of participants in the current study had seen rock hyraxes in their localities and over one third of them also responded that they used hyrax dung as a fertilizer in their farm plots. Hyrax species have been shown to be the primary reservoir hosts of CL parasites in Ethiopia [45] and two studies conducted in Tigray have indicated that the presence of hyraxes close to resident houses was significantly associated with CL infections [15,46]. However, only a very small number of our participants were informed regarding hyraxes as potentially involved in CL transmission.

Considering the present findings, the communities living in CL-endemic areas of Tigray do not have adequate information on prevention and control of the disease. Providing more education on CL transmission, the insect vector (sandfly), the reservoir hosts (hyrax), and activities/times that are associated with a greater risk of CL would help communities to take preventive measures against the disease.

### 4.6. Limitations of the Current Study

This study included participants from seven districts within Tigray and may not capture localized differences in knowledge, attitudes, treatment-seeking behaviours, and prevention practices, although it provides an overall regional perspective. The findings may also not be generalizable to the wider population of Ethiopia. As this was a cross-sectional study, recall bias is a potential limitation, and social desirability bias may have influenced responses, particularly for questions related to treatment-seeking behaviours.

The categorisation of participants into “good” and “poor” knowledge and “positive” and “negative” attitudes and practices enabled the identification of key associated factors, notably the strong association between prior household CL experience and improved knowledge. However, this approach may mask important nuances, including very low levels of understanding of disease transmission. In addition, knowledge and attitudes towards CL are shaped by multiple interacting social and contextual factors that were not fully captured in this study and require more in-depth investigation.

Therefore, the findings should be interpreted with caution. This work forms part of the wider ECLIPSE program, and further insights from ongoing qualitative research will help to contextualize and extend these findings.

## 5. Conclusions

In this study, more than half of participants from a CL-endemic region of Tigray had an overall poor level of knowledge about the disease. While the local name (Guzwa) and the main clinical signs were widely recognized, understanding of disease transmission, including the sand fly vector and reservoir host, was very limited, with likely implications for prevention efforts. More than two-thirds of participants exhibited unfavourable attitudes towards CL, and a majority perceived the disease to be highly stigmatizing.

Most participants also had a poor understanding of available biomedical treatments, with traditional methods remaining the primary care choice. In addition, prevention and treatment-seeking practices were generally suboptimal. Occupation and household setting were significantly associated with attitudes, while gender, education level, and prior household CL experience were key determinants of treatment-seeking and prevention practices.

These findings highlight a disconnect between high disease burden, perceived seriousness and stigma, and limited knowledge and appropriate health behaviours. They reflect a lack of effective CL awareness and prevention programs in the region and underscore the need for targeted, community-based interventions. In particular, improved health education focusing on transmission, prevention measures, and access to effective treatment, alongside strategies to address stigma and structural barriers to care, will be essential to support CL control in affected communities.

## Figures and Tables

**Table 1:** Socio-demographic characteristics of study participants in Tigray, North Ethiopia (*n* = 512).

Variable	Categories	Frequency (*n*)	Percent (%)
Zone	Eastern	268	52.3
	South Eastern	142	27.7
	Southern	102	20.0
Districts	Degua Temben	94	18.4
	Emba-alaje	102	19.9
	Enderta	48	9.4
	Ganta Afeshum	56	10.9
	Gulomekeda	41	8.0
	Hawzen	70	13.7
	Saesie Tsaeda Emba	101	19.7
Household setting	Rural	375	73.2
	Urban/semi-urban	137	26.8
Age group	18–40	361	70.5
	≥41	151	29.5
Gender	Male	268	52.3
	Female	244	47.7
Educational background	Formal school unattended	154	30.1
	Primary school (1–6)	202	39.4
	Secondary school (7–10)	149	29.1
	College & above	7	1.4
Primary occupation	Daily labourer	34	6.6
	Farmer	406	79.3
	Trade/merchant	26	5.1
	Government employee	20	3.9
	Student	26	5.1
CL episode in household in the last five years?	Yes	312	60.9
	No	200	39.1

**Table 2:** Knowledge about cutaneous leishmaniasis (CL), clinical presentations, transmission, vector, and reservoir hosts among communities in the Tigray region.

Questions	Responses	Frequency	Percent (%)
Have you seen or heard about the disease ^®^?	Yes *	498	97.3
	No	14	2.7
Main source of information?	Household family members with experience of CL	256	51.4
	Neighbours with experience of CL	226	45.4
	Mass media (TV, radio)	6	1.2
	Traditional healer	6	1.2
	Healthcare facility	4	0.8
What do you call the disease?	Local name (Guzwa)	482	96.8
	Leishmaniasis	16	3.2
Symptoms of CL ^®^	Skin lesions *	356	71.5
	Lasting skin wound	72	14.5
	Skin scar	70	14.0
Mostly affected body parts ^®^?	Face *	474	95.2
	Hands	116	23.3
	Legs	48	9.6
How is CL transmitted ^®^?	Bat (feces or urine)	161	32.3
	Genetically from family	124	24.9
	Butterfly (“Tsa/Guzwa”)	87	17.5
	Contact with CL infected	8	1.6
	Mosquito bite	7	1.4
	Sand fly bite *	6	1.2
	Don’t know	105	21.1
What is the season for peak CL infections ^®^?	In all seasons equally	127	25.5
	Dry season	41	8.2
	Post rainy season *	224	45.0
	Don’t know	106	21.3
Where do people seek CL treatment?	Formal healthcare	45	9.0
	Traditional remedies	313	62.9
	Faith (religious) healer	70	14.1
	Don’t know	70	14.1
Do you know about biomedical CL treatment ^®^?	Yes *	149	29.9
	No	13	2.6
	Don’t know	336	67.5
Know or seen rock hyrax in your locality ^®^?	Yes *	376	73.6
	No	135	26.4
Can rock hyrax be an agent for CL infection ^®^?	Yes *	12	3.2
	No	20	5.3
	Don’t know	344	91.5
Is CL preventable ^®^?	Yes *	140	27.3
	No	9	1.8
	Don’t know	349	68.2
Overall knowledge about CL			
Total mean score ± SD		5.3 ± 1.4	
Good knowledge (score 5–9)		219	42.8
Poor knowledge (score 0–4)		293	57.2

^®^ Items included in scoring. * Correct responses were assigned a score of 1 and other responses were assigned 0.

**Table 3:** Attitudes toward cutaneous leishmaniasis (CL), its treatment, and care provider priorities of community members in the Tigray region.

Questions/Responses		Frequency	Percent (%)
Is CL a serious health concern in your locality ^®^?	Yes *	478	96.0
	No	20	4.0
Can CL be genetically acquired from family?	Yes	124	24.9
	No *	247	49.6
	Don’t know	127	25.5
Is CL a stigmatizing disease ^®^?	Yes	335	67.3
	No *	13	2.6
	Don’t know	150	30.1
Do you feel bad when meeting people with CL lesion ^®^?	Yes	446	87.1
	No *	52	10.2
	Not sure	14	2.7
Is CL curable by treatments ^®^?	Yes *	437	87.8
	No	10	2.0
	Don’t know	51	10.2
Is it possible to prevent CL infection ^®^?	Yes *	140	27.3
	No	9	1.8
	Don’t know	349	68.2
Where is your best care provider choice?	Formal healthcare	43	10.0
	Traditional healer (herbalist)	315	73.6
	Faith healers (Religious)	70	16.4
Do you want to seek formal healthcare for CL?	Yes	149	29.9
	No	62	12.4
	Not sure	287	57.6
Overall attitude status			
Total mean score ± SD		3.2 ± 1.1	
Positive attitude (score 4–6)		187	36.5
Negative attitude (score 1–3)		325	63.5

^®^ Items included in scoring; * correct responses were assigned a score of 1 and other responses were assigned 0.

**Table 4:** Treatment-seeking behaviours and practices of community members in the Tigray region.

Questions	Responses	Frequency	Percent (%)
Communities’ primary form of care for active CL	Homemade herbals	425	83.0
	Traditional healer (herbalist)	315	63.3
	Using hot iron (cauterization)	89	17.4
	Religious faith healer	70	13.7
	Formal healthcare facility (hospital/clinic)	45	9.0
	Do nothing (self-curable)	12	2.3
Main reason to choose care provider?	Proximity to home	243	48.8
	Perceived (good) reputation	148	29.7
	Availability of CL drugs	37	7.4
	Other reasons	70	14.1
Have your household ever used a formal CL healthcare facility?	Yes	136	27.3
	No	362	72.7
During the last CL episode in your household, how was the lesion finally managed (*n* = 312)?	Using biomedical treatment in hospital	136	43.6
	Using traditional remedies	67	21.5
	Self-cured (did nothing)	95	30.4
	Lesion persisted, has not cured yet	14	4.5
How many days later received formal healthcare (*n* = 136)	<30 days	13	9.6
	31–180 days	91	66.9
	181–365 days	12	8.8
	>365 days	20	14.7
Distance to formal healthcare facility (Km) from home?	<30 km	11	7.4
	31–60 km	40	26.8
	61–90 km	90	60.4
	>91 km	8	5.4
When do people seek modern treatment for CL?	For “male” type CL	237	47.6
	For “runner” type CL	37	7.4
	When traditional care failed to resolve the lesion	78	15.7
	Don’t know	146	29.3

**Table 5:** Common activities and prevention practices of community members in the Tigray region.

Field work during both day and night?	Yes	118	23.1
	No *	394	76.9
Field work from early morning until late evening?	Yes	232	45.3
	No *	280	54.7
Outdoor/field/sleeping?	Yes	298	58.2
	No *	214	41.8
Use of hyrax dung as fertilizer	Yes	194	37.9
	No *	318	62.1
Did your household ever receive formal CL care?	Yes *	136	27.8
	No	353	72.2
Before receiving a modern CL care, did your household use other care options (*n* = 136)?	Yes	115	84.6
	No *	21	15.4
Use prevention methods (*n* = 489)?	Yes *	140	28.6
	No	349	71.4
Sleep under a bed net	Yes *	138	28.2
	No	351	71.8
Environmental hygiene	Yes *	52	10.6
	No	437	89.4
Wear long sleeves/trousers	Yes *	36	7.4
	No	453	92.6
Insecticide spray in house	Yes *	34	7.0
	No	455	93.0
Use of insect repellents	Yes *	11	2.2
	No	478	97.8
Overall treatment-seeking behaviours and prevention practices level			
Total mean score ± SD	–	4.48 ± 2.02	
Good (score 7–12)	–	173	33.8
Poor (score 0–6)	–	339	66.2

* Correct/positive responses were assigned a score of 1. Other responses were assigned 0.

**Table 6:** Demographic factors compared with participants’ knowledge about cutaneous leishmaniasis (CL) in the Tigray region.

Variables and Categories	Knowledge About CL		COR (95% CI)	AOR (95% CI)	*p* Value AOR
	Good	Poor			
Gender					
Male	124 (46.3%)	144 (53.7%)	0.74 (0.52, 1.05)	0. 55 (0.36, 0.83)	0.004
Female	95 (38.9%)	149 (61.1%)	1	1	
Age group (year)					
18–40	155 (42.9%)	206 (57.1%)	1.02 (0.70, 1.50)	0.85 (0.52, 1.38)	0.507
>40	64 (42.4%)	87 (57.6%)	1	1	
Educational background					
Educated	70 (45.5%)	84 (54.5%)	1.17 (0.80, 1.71)	0.79 (0.48, 1.29)	0.354
Formal school unattended	149 (41.6%)	209 (58.4%)	1	1	
Primary occupation					
Farmer	183 (45.1%)	223 (54.9%)	1.60 (1.02, 2.50)	0.53 (0.31, 0.91)	0.021
Non-farmer	36 (34.0%)	70 (66.0%)	1	1	
Household setting					
Rural	155 (41.3%)	220 (58.7%)	0.80 (0.54, 1.19)	1.60 (1.00, 2.57)	0.049
Urban or semi-urban	64 (46.7%)	73 (53.3%)	1	1	
Previous or current CL episode in household					
Yes	190 (69.9%)	122 (39.1%)	0.11 (0.07, 0.17)	10.19 (6.36, 16.30)	<0.001
No	29 (14.5%)	171 (85.5%)	1	1	

**Table 7:** Factors associated with study participants’ attitudes towards cutaneous leishmaniasis (CL) among community members in the Tigray region.

Variables and Categories	Attitude About CL		COR (95% CI)	AOR (95% CI)	*p* Value for AOR
	Negative	Positive			
Gender					
Male	92 (34.3%)	176 (65.7%)	1.22 (0.85, 1.77)	1.18 (0.81, 1.71)	0.392
Female	95 (38.9%)	149 (61.1%)	1	1	
Age group (year)					
18–40	136 (37.7%)	225 (62.3%)	1.18 (0.79, 1.76)	0.73 (0.47, 1.14)	0.165
>40	51 (33.8%)	100 (66.2%)	1	1	
Educational background					
Educated	128 (35.8%)	230 (64.2%)	1.12 (0.75, 1.65)	0.79 (0.50, 1.24)	0.310
Formal school unattended	59 (38.3%)	95 (61.7%)	1	1	
Primary occupation					
Farmer	155 (38.2%)	251 (61.8%)	1.43 (0.90, 2.26)	0.60 (0.36, 0.99)	0.048
Non-farmer	32 (30.2%)	74 (69.8%)	1	1	
Household setting					
Rural	122 (32.5%)	253 (67.5%)	0.53 (0.36, 0.80)	2.17 (1.42, 3.31)	<0.001
Urban/semi-urban	65 (47.4%)	72 (52.6%)	1	1	
Previous or current CL episode in household					
Yes	123 (39.4%)	189 (60.6%)	0.72 (0.50, 1.05)	1.37 (0.94, 2.01)	0.102
No	64 (32.0%)	136 (68.0%)	1	1	

**Table 8:** Factors associated with participants’ treatment-seeking and prevention practices for cutaneous leishmaniasis (CL) in the Tigray region.

Variables and Categories	Treatment-Seeking and Prevention Practices		COR (95% CI)	AOR (95% CI)	*p* Value for AOR
	Good	Poor			
Gender					
Male	79 (29.5%)	189 (70.5%)	1.22 (0.85, 1.75)	1.49 (1.01, 2.22)	0.044
Female	94 (38.5%)	150 (61.5%)	1	1	
Age group (year)					
18–40	112 (31.0%)	249 (69.0%)	1.18 (0.79, 1.76)	1.01 (0.63, 1.59)	0.982
>40	61 (40.4%)	90 (59.6%)	1	1	
Educational background					
Educated	73 (47.4%)	81 (52.6%)	1.12 (0.75, 1.65)	0.39 (0.24, 0.62)	<0.001
Formal school unattended	100 (27.9%)	258 (72.1%)	1	1	
Primary occupation					
Farmer	139 (34.2%)	267 (65.8%)	1.43 (0.90, 2.26)	1.52 (0.91, 2.54)	0.112
Non-farmer	34 (32.1%)	72 (67.9%)	1	1	
Household setting					
Rural	136 (36.3%)	239 (63.7%)	0.53 (0.36, 0.80)	0.67 (0.42, 1.06)	0.086
Urban/semi-urban	37 (27.0%)	100 (73.0%)	1	1	
Previous/current CL episode in household					
Yes	132 (42.3%)	180 (57.7%)	0.72 (0.50, 1.05)	2.99 (1.96, 4.57)	<0.001
No	41 (20.5%)	159 (79.5%)	1	1	

## Data Availability

Datasets used in this study are available from the corresponding author on reasonable request.

## Abbreviations

The following abbreviations are used in this manuscript:

AORs	Adjusted odd ratios
CIs	Confidence intervals
CL	Cutaneous leishmaniasis
DCL	Diffuse cutaneous leishmaniasis
EAs	Enumeration areas
HH	Household
MCL	Mucocutaneous leishmaniasis

## References

[1] Burza S., Croft S.L., Boelaert M. (2018). Leishmaniasis. Lancet.

[2] Alvar J., Velez I.D., Bern C., Herrero M., Desjeux P., Cano J., Jannin J., Boer M.D., WHO Leishmaniasis Control Team (2012). Leishmaniasis worldwide and global estimates of its incidence. PLoS ONE.

[3] World Health Organization (WHO) Leishmaniasis 2023: Key Facts. https://www.who.int/news-room/fact-sheets/detail/leishmaniasis.

[4] CDC (2020). Leishmaniasis: Epidemiology & Risk Factors.

[5] Shita E.Y., Semegn E.N., Wubetu G.Y., Abitew A.M., Andualem B.G., Alemneh M.G. (2022). Prevalence of Leishmania RNA virus in Leishmania parasites in patients with tegumentary leishmaniasis: A systematic review and meta-analysis. PLoS Negl. Trop. Dis..

[6] Bailey F., Mondragon-Shem K., Hotez P., Ruiz-Postigo J.A., Al-Salem W., Acosta-Serrano A., Molyneux D.H. (2017). A new perspective on cutaneous leishmaniasis-Implications for global prevalence and burden of disease estimates. PLoS Negl. Trop. Dis..

[7] Nuwangi H., Agampodi T.C., Price H.P., Shepherd T., Weerakoon K.G., Agampodi S.B. (2023). Stigma associated with cutaneous and mucocutaneous leishmaniasis: A systematic review. PLoS Negl. Trop. Dis..

[8] World Health Organization (2022). Global Health Observatory Data Repository.

[9] Al Ashwal M., Al Adhroey A., Atroosh W., Alshoteri S., Al Subbary A., Alharazi T., Sady H., Azzani M., Lau Y.L., Al-Mekhlafi H.M. (2024). Knowledge, attitude, practices and treatment seeking behaviour concerning cutaneous leishmaniasis among rural hyperendemic communities in western Yemen. Sci. Rep..

[10] Poggi I. (1937). Oriental sore in Agamé (Abyssinia). Arch. Ital. Sci. Med. Trop. Parasitol..

[11] Ethiopian Federal Ministry of Health (2013). National Master Plan for Neglected Tropical Disease (2013–2015).

[12] van Henten S., Adriaensen W., Fikre H., Akuffo H., Diro E., Hailu A., Van der Auwera G., van Griensven J. (2018). Cutaneous leishmaniasis due to Leishmania aethiopica. eClinicalMedicine.

[13] Doni S., Yeneneh K., Hailemichael Y., Gebremichael M., Skarbek S., Ayele S., Tadesse A.W., Lambert S., Walker S.L., Gadisa E. (2023). Health-related quality of life of adults with cutaneous leishmaniasis at ALERT Hospital, Addis Ababa, Ethiopia. PLoS Negl. Trop. Dis..

[14] Shita E.Y., Nibret E., Munshea A., Gashaw B. (2022). Burden and risk factors of cutaneous Leishmaniasis in Ethiopia: A systematic review and Meta-Analysis. Int. J. Dermatol..

[15] Tessema S.B., Hailu B.G., Embaye T.H., Debes B.F., Nigus G.K., Reda B.S., Price H.P., Bezabih A.M. (2026). Cutaneous Leishmaniasis in Tigray, Ethiopia: Clinical patterns, environmental drivers and public health implications. PLoS Negl. Trop. Dis..

[16] Alharazi T.H., Haouas N., Al-Mekhlafi H.M. (2021). Knowledge and attitude towards cutaneous leishmaniasis among rural endemic communities in Shara’b district, Taiz, southwestern Yemen. BMC Infect. Dis..

[17] Alidosti M., Heidari Z., Shahnazi H., Zamani-Alavijeh F. (2021). Behaviors and perceptions related to cutaneous Leishmaniasis in endemic areas of the world: A review. Acta Trop..

[18] Kebede N., Worku A., Ali A., Animut A., Negash Y., Gebreyes A., Satoskar A. (2016). Community knowledge, attitude and practice towards cutaneous leishmaniasis endemic area Ochello, Gamo Gofa Zone, South Ethiopia. Asian Pac. J. Trop. Biomed..

[19] Pardo R.H., Carvajal A., Ferro C., Davies C.R. (2006). Effect of knowledge and economic status on sandfly control activities by householders at risk of cutaneous leishmaniasis in the sub-Anden region of Huila Department, Colombia. Biomedica.

[20] Alemayehu B., Kelbore A.G., Alemayehu M., Adugna C., Bibo T., Megaze A., Leirs H. (2023). Knowledge, attitude, and practice of the rural community about cutaneous leishmaniasis in Wolaita zone, southern Ethiopia. PLoS ONE.

[21] Tamiru H., Mashalla Y., Mohammed R., Tshweneagae G. (2019). Cutaneous leishmaniasis a neglected tropical disease: Community knowledge, attitude and practice in an endemic area, Northwest Ethiopia. BMC Infect. Dis..

[22] Berhanu A., Dugassa S., Maru M., Animut A., Erko B., Hailu A., Gebresilassie A. (2023). Cutaneous leishmaniasis in Kutaber District, Ethiopia: Prevalence, sand fly fauna and community knowledge, attitude and practices. Heliyon.

[23] Lwanga S.K., Lemeshow S. (1991). Sample Size Determination in Health Studies: A Practical Manual.

[24] Central Statistical Agency [Ethiopia] and ICF International (2012). Ethiopia Demographic and Health Survey 2011.

[25] Akram A., Ali Khan H.A., Qadir A., Sabi A.M. (2015). A cross-sectional survey of knowledge, attitude and practice related to cutaneous leishmaniasis and sand flies in Punjab, Pakistan. PLoS ONE.

[26] Dires A., Kumar P., Gedamu S., Yimam W., Ademe S. (2022). Determinants of cutaneous leishmaniasis among students in Delanta district, Northeast Ethiopia: A case-control study. Health Sci. Rep..

[27] Doe E.D., Egyier-Yawson A., Kwakye-Nuako G. (2019). Knowledge, attitude and practices related to cutaneous leishmaniasis in endemic communities in the Volta region of Ghana. Int. J. Healthc. Sci..

[28] Ali R.M., Loutfy N.F., Awad O.M. (2015). Attitude and knowledge of primary health care physicians and local inhabitants about leishmaniasis and sand fly in West Alexandria. Int. J. Hum. Soc. Sci..

[29] Saberi S., Zamani A., Motamedi N., Nilforoushzadeh M.A., Jaffary F., Rahimi E., Hejazi S.H. (2012). The knowledge, attitude, and prevention practices of students regarding cutaneous leishmaniasis in the hyperendemic region of the Shahid Babaie Airbase. Vector Borne Zoonotic Dis..

[30] Gunasekara S.D., Agampodi T.C., Weerasinghe M., Fernando M.S., Price H.P., Wickramasinghe N.D., Agampodi S.B. (2024). Investigating disease awareness of cutaneous leishmaniasis in rural Sri Lanka to inform public health services: A cross-sectional study. BMJ Open.

[31] Wijesinghe H., Gunathilaka N., Semege S., Pathirana N., Manamperi N., de Silva C., Fernando D. (2020). Histopathology of Cutaneous Leishmaniasis Caused by Leishmania donovani in Sri Lanka. BioMed Res. Int..

[32] Nuwangi H., Dikomitis L., Weerakoon K.G., Liyanage C., Agampodi T.C., Agampodi S.B. (2024). Stigma associated with cutaneous leishmaniasis in rural Sri Lanka: Development of a conceptual framework. Int. Health.

[33] Grifferty G., Shirley H., McGloin J., Kahn J., Orriols A., Wamai R. (2021). Vulnerabilities to and the Socioeconomic and Psychosocial Impacts of the Leishmaniases: A systematic Review. Res. Rep. Trop. Med..

[34] Al-Kamel M.A. (2017). Stigmata in cutaneous leishmaniasis: Historical and new evidence-based concepts. Our Dermatol. Online.

[35] Boukthir A., Bettaieb J., Erber A.C., Bouguerra H., Mallekh R., Naouar I., Gharbi A., Alghamdi M., Plugge E., Olliaro P. (2020). Psycho-social impacts, experiences and perspectives of patients with Cutaneous Leishmaniasis regarding treatment options and case management: An exploratory qualitative study in Tunisia. PLoS ONE.

[36] Bennis I., Belaid L., De Brouwere V., Filali H., Sahibi H., Boelaert M. (2017). The mosquitoes that destroy your face. Social impact of Cutaneous Leishmaniasis in South-eastern Morocco, A qualitative study. PLoS ONE.

[37] Bennis I., Thys S., Filali H., De Brouwere V., Sahibi H., Boelaert M. (2017). Physchosocial impact of scars due to cutaneous leishmaniasis in high school students in Errachidia province, Morocco. Infect. Dis. Poverty.

[38] Chahed M.K., Bellali H., Ben Jemaa S., Bellaj T. (2016). Psychological and Psychosocial Consequences of Zoonotic Cutaneous Leishmaniasis among Women in Tunisia: Preliminary Findings from an Exploratory Study. PLoS Negl. Trop. Dis..

[39] Wenning B., Price H., Nuwangi H., Reda K.T., Walters B., Ehsanullah R., Viana G., Andras A., Dikomitis L. (2022). Exploring the cultural effects of gender on perceptions of cutaneous leishmaniasis: A systematic literature review. Glob. Health Res. Policy.

[40] Torbosh A., Al Amad M.A., Al Serouri A., Khader Y. (2019). The impact of war in Yemen on immunization coverage of children under one year of age: Descriptive study. JMIR Public Health Surveill..

[41] Gesesew H., Berhane K., Siraj E.S., Siraj D., Gebregziabher M., Gebre Y.G., Gebreslassie S.A., Amdeslassie F., Tesema A.G., Siraj A. (2021). The impact of war on the health system of the Tigray region in Ethiopia: An assessment. BMJ Glob. Health.

[42] Tessema S.B., Hagos T., Kehasy G., Paintain L., Adera C., Herrero M., den Boer M., Temesgen H., Price H., Mulugeta A. (2024). The economic burden of visceral leishmaniasis and barriers to accessing healthcare in Tigray, North Ethiopia: A field based study. PLoS Negl. Trop. Dis..

[43] Okeke T.A., Okafor H.U., Uzochukwu B.S. (2006). Traditional healers in Nigeria: Perception of cause, treatment and referral practices for severe malaria. J. Biocos Sci..

[44] Bantie B., Kassaw G., Demelash A.T., Abate M.W., Nigat A.B., Amare A.T., Birlie T.A., Tasew S.F., Zeleke S., Kassie A. (2024). Magnitude and associated factors of cutaneous leishmaniasis among patients visiting Nefas Mewcha primary hospital, Northern Ethiopia, 2022: An institution-based Crosssectional study. BMJ Open.

[45] Jemberie W., Animut A., Dugassa S., Gebresilassie A., Melkamu R., Aklilu E., Aemero M., van Griensven J., Pareyn M. (2024). Ecology and Infection Status of Sand Flies in Rural and Urban Cutaneous Leishmaniasis Endemic Areas in Northwest Ethiopia. Trop. Med. Infect. Dis..

[46] Bsrat A., Berhe N., Balkew M., Yohannes M., Teklu T., Gadisa E., Medhin G., Abera A. (2015). Epidemiological study of cutaneous leishmaniasis in Saesie Tsaeda-emba district, eastern Tigray, northern Ethiopia. Parasit. Vectors.

